# Percutaneous Ethanol Ablation of Parathyroid Adenoma and Parathyroidectomy Are Comparable

**DOI:** 10.1155/ije/4939126

**Published:** 2026-07-05

**Authors:** Iraj Heydari, Sara Golmohammadi, Mohammad Reza Babaei, Hamed Iraji, Hossein Akhondi

**Affiliations:** ^1^ Endocrine Research Center, Institute of Endocrinology and Metabolism, Iran University of Medical Sciences, Tehran, Iran, iums.ac.ir; ^2^ School of Medicine, Iran University of Medical Sciences, Tehran, Iran, iums.ac.ir; ^3^ Interventional Radiology, Firouzgar Hospital, Iran University of Medical Sciences, Tehran, Iran, iums.ac.ir; ^4^ Graduate Medical Education, University of Nevada, Reno, Nevada, USA, unlv.edu

## Abstract

**Aim:**

To compare ethanol injection of parathyroid adenoma with surgical parathyroidectomy (PTX).

**Methods:**

A prospective, partially randomized preference clinical trial of two groups assigned to surgical PTX (20 patients) or ethanol injection (PEA) (10 patients). The inequality was due to patient preferences and limitations caused by the coronavirus pandemic.

**Results:**

Both PEA and PTX resulted in 80% or more of patients having normalized calcium levels after one month. These were sustained through 6 months. A significant proportion of patients after both PEA and PTX had normalized PTH levels after one month (55% and 70%, respectively), which remained unchanged at the 3‐ and 6‐month measurements. After 12 months, calcium and parathyroid hormone levels were lower in the PTX group, while phosphorus levels were higher; however, the differences were not statistically significant. Complications were more common in the PTX group, although the difference was not statistically significant.

**Conclusion:**

Overall, PEA and PTX have similar outcomes. Although not statistically significant, the PEA of parathyroid adenoma causes a less pronounced effect on calcium and parathyroid hormone levels and has fewer side effects.

## 1. Introduction

Hyperparathyroidism occurs due to increased activity of parathyroid glands. This condition is caused by intrinsic problems within the parathyroid glands, called primary or tertiary hyperparathyroidism. It may also be caused by a problem outside the parathyroid glands that affects calcium homeostasis and stimulates parathyroid hormone (PTH) production, a condition known as secondary hyperparathyroidism. Primary hyperparathyroidism is the third most common endocrine disease and the leading cause of hypercalcemia, with a prevalence of 0.1% to 0.4%. Parathyroid adenoma is the primary reason (85%–90%). Most patients are asymptomatic and are incidentally diagnosed with hypercalcemia during a laboratory examination. Patients with symptomatic disease can still have kidney stones, skeletal complications, and osteoporosis with fractured bones [1–5]. In primary hyperparathyroidism, ionized serum calcium is elevated, and the serum phosphorus level is usually within the lower half of the normal range (0.97–1.13 mmol/L). The presence of increased or inappropriately normal PTH levels (PTH ≥ 20 ng/L with hypercalcemia) confirms the diagnosis of hyperparathyroidism [6–8]. Adenomas can be located using ultrasound, magnetic resonance imaging, scintigraphy, and single‐photon emission computed tomography (SPECT) scans with technetium‐99 [9, 10]. Surgical parathyroidectomy (PTX) is the standard treatment for primary hyperparathyroidism [11, 12]. Medical treatment is required if there are contraindications to PTX or the patient is not interested in surgical options [13, 14].

A surgical procedure can result in complications, including infection, hematoma, permanent hypoparathyroidism, adhesion of muscles to the larynx or trachea, damage to the recurrent laryngeal nerve, problems during anesthesia, and the formation of keloid scars. Furthermore, there is a 4%–10% chance of recurrence of hypercalcemia after surgical treatment [15]. A relapse or failure to respond to the medical treatment is also possible [16, 17].

Solbiati et al. used nonsurgical methods to treat hyperparathyroidism for the first time in 1985 [18]. In recent years, minimally invasive treatment methods have been used more frequently, including ethanol injection into the adenoma, radiofrequency (RF) ablation, and microwave ablation (MWA), all having fewer complications than surgical PTX [1–5].

Percutaneous ethanol ablation (PEA) is more cost‐effective, accessible, and repeatable than the other methods. If it fails, other options are still available, including minimally invasive MWA, RF ablation, and PTX. The side effects associated with ethanol injection include recurrent laryngeal nerve injury and pain at the injection site that may radiate to the jaw and teeth on the same side. Such pain resolves after the injection. Recurrent laryngeal nerve injury can also occur in 0.5%–2% of surgeries that an experienced surgeon performs [19].

Researchers have conducted several studies on the effectiveness of different treatment methods, and the results have been contradictory and variable, depending on the technician’s skills, the procedure’s process, and other factors. Neither of these methods has been proposed as a standard or definitive treatment for hyperparathyroidism, and PTX is still the standard treatment. Therefore, more studies are needed to determine the correct course of action.

We conducted a partially randomized preference clinical trial involving patients with parathyroid adenoma, randomly assigned to PTX and PEA. Patient preference changes the grouping. We measured the levels of calcium, phosphorus, and PTH following the therapeutic intervention. We compared the effectiveness of the therapeutic methods between the two groups. The team also monitored for complications at each visit and after each injection. One unique feature of this study is that patients who were suitable candidates for PTX and could have received it were assigned to the PEA group, based on randomization or patient preference.

## 2. Materials and Methods

The Institutional Review Board of Iran University of Medical Sciences approved this study (Code of ethics: IR.IUMS.FMD.REC.1399.722). We obtained informed consent from all participants who expressed their desire to participate in the research. The team also fully informed the patients of the benefits and possible risks associated with each treatment method. The researchers fully disclosed all study details. Study was carried out in Iran and not registered with CTRI.

This trial evaluated patients with primary hyperparathyroidism at the Endocrine Institute of Firouzgar Hospital in Tehran/Iran. The inclusion criteria were the presence of parathyroid adenoma and an age of more than 18. Exclusion criteria included chronic obstructive pulmonary disease, lesions in proximity to the cervical ganglia and recurrent laryngeal nerve, significant orthostatic hypotension, ectopic parathyroid adenoma (other than neck), and malignancy of the parathyroid glands.

The patient’s medical history, including a history of kidney stones, osteoporosis, underlying diseases, and surgical history, was taken. We tested the patients for serum calcium, phosphorus, PTH, and creatinine. An analyzer device (Hitachi) measured serum calcium and phosphorus levels, while another analyzer (Auto Bio) device measured PTH levels. Vit D and coagulation panels were not checked. Our radiologists also performed a neck ultrasound to determine the number, location, and size of the parathyroid adenoma(s). Although other methods such as radionuclide scan, SPECT, or regular computed tomography have been used for localization at times, they were not used so that ultrasound remains the sole method of localization for all cases. Primary hyperparathyroidism was diagnosed based on a serum calcium level greater than 10.5 mg/dL, a serum PTH level greater than 65 ng/L, and a parathyroid adenoma on ultrasound. Afterward, we randomly assigned the patients to one of the two treatment groups: PTX or PEA. However, if they preferred a specific treatment, they were assigned to that group, unless there was a clear contraindication for the procedure in question. This process resulted in unequal groups, as more patients chose the surgical method. Parathyroid malignancy and multiple endocrine neoplasia Type1 were ruled out based on pathology and history and were excluded from the study.

We evaluated both groups of patients at 1, 3, and 6 months following the procedure for calcium, phosphorus, and PTH serum levels, voice hoarseness, hypocalcemic symptoms, kidney stones, and recurrence of symptoms. Additionally, we examined six patients one year later and four patients two years later. The PTX group consisted of 20 patients (11 women and nine men) with an average age of 52, who underwent PTX by a single surgeon. Standard PTX was performed by a single surgeon. No intraoperative PTH monitoring was available.

**FIGURE 1:**
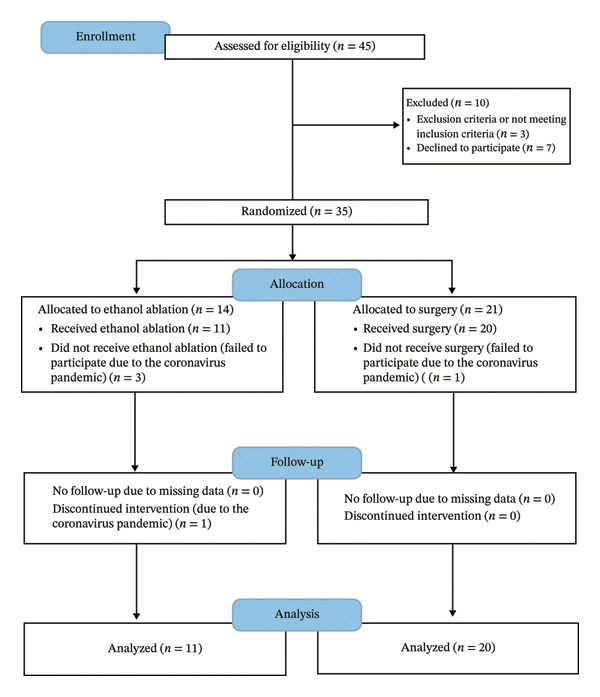
CONSORT diagram.

A total of 14 patients were included in the group that received ethanol injections. Despite requests, three failed to participate due to the coronavirus pandemic. Ethanol injection into the adenoma was performed on 11 patients (4 women and 7 men) whose average age was 47.18 years. One of the patients dropped out after the first injection and, therefore, yielded incomplete results (Figure 1). A single radiologist administered 0.5–2 cc of 99.6% ethanol, depending on the size of the adenoma, under ultrasound guidance with a 22‐gauge needle in several areas of the adenoma. Subjects were discharged from the hospital an hour after the procedure. If there was no response after one month, the radiologists repeated the ethanol injections. Each patient received an average of 2.09 (SD ± 1.3) ethanol injections. After the injections, if the patient’s tests remained abnormal, they were referred for PTX.

A patient with adenomas in two parathyroid glands required five injections. Four of the 31 patients had two parathyroid adenomas (2 in the PEA group and 2 in the PTX group).

The statistician analyzed the data using SPSS Statistics software, Version 26. Quantitative variables were expressed as mean ± standard deviation. Qualitative values were analyzed using chi‐square and paired *t*‐test. The confidence interval of the data was considered statistically significant at *p* < 0.05. The statistician conducted a univariate analysis using the Chi‐square test to compare the two groups with respect to sex, age, underlying diseases, history of prior minor surgery, and adenoma size.

## 3. Results

**FIGURE 2:**
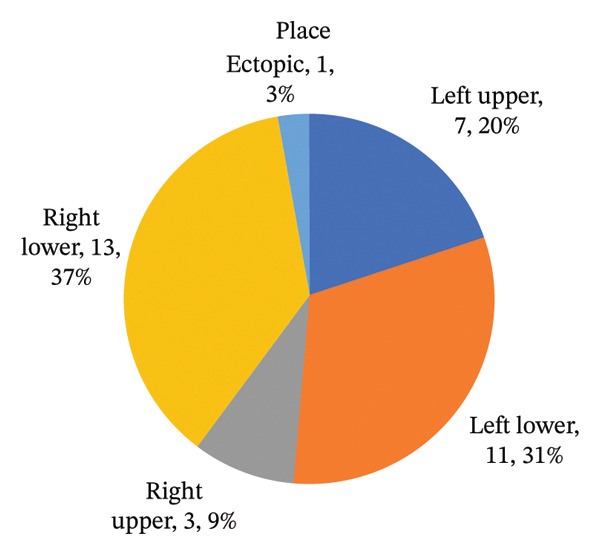
Distribution of adenomas.

Table 1 presents the demographics of the patients. Figure 2 shows the distribution of adenomas’ locations. Table 2 presents the laboratory test results for the PTX and PEA groups before and after the procedure. A paired *t*‐test was conducted to compare the values between the two groups.

**TABLE 1:** Study demographics for parathyroidectomy (PTX) and percutaneous ethanol ablation (PEA).

	**PTX**	**PEA**	**p** **value**	

Age	52 ± 13.1	47.18 ± 9.89	0.483	T‐test
Female number	11	4	0.458	T‐test
Female percent	55%	36%	0.458	T‐test
Male number	9	7	0.458	T‐test
Male percent	45%	64%	0.458	T‐test
Diabetes number	6	4	1	T‐test
Diabetes percent	30%	36%	1	T‐test
Hypertension number	8	3	0.698	T‐test
Hypertension percent	40%	27%	0.698	T‐test
Heart ischemia number	0	1	0.355	T‐test
Heart ischemia percent	0%	9%	0.355	T‐test
Prior minor PTX number	7	3	1	T‐test
Prior Minor PTX percent	35%	27%	1	T‐test
Adenoma length and width	96.03 ± 50.12	97.35 ± 75.08	0.963	T‐test

**TABLE 2:** Lab values of calcium (Ca), parathormone (PTH), and phosphorus (P) in the PEA and PTX groups at baseline and Months 1, 3, and 6.

	**Ca (mg/dL)**	**PTH (pg/mL)**	**P (mg/d)**	**Ca (mg/dL)**	**PTH (pg/m)**	**P (mg/dL)**	**Ca (mg/dL)**	**PTH (pg/m)**	**P (mg/dL)**	**Ca (mg/dL)**	**PTH (pg/mL)**	**P (mg/dL)**

PEA	11.45 ± 0.73	170.39 ± 74.58	2.97 ± 0.7	9.85 ± 0.92	92.89 ± 63.71	3.28 ± 0.88	9.63 ± 0.9	91 ± 64.55	3.2 ± 0.98	9.75 ± 0.94	86 ± 49.92	3.37 ± 0.88
PTX	11.46 ± 1	149 ± 63.09	3.15 ± 0.72	9.36 ± 0.96	66.04 ± 37.71	3.56 ± 0.63	9.38 ± 0.77	64.5 ± 34.33	3.66 ± 0.53	9.29 ± 0.88	63 ± 38.69	3.66 ± 0.48
Statistical test	Paired *t*‐test	Paired *t*‐test	Paired *t*‐test	Paired *t*‐test	Paired *t*‐test	Paired *t*‐test	Paired *t*‐test	Paired *t*‐test	Paired *t*‐test	Paired *t*‐test	Paired *t*‐test	Paired *t*‐test
*p* value	0.976	0.403	0.519	0.186	0.149	0.321	0.422	0.143	0.093	0.186	0.163	0.252
	M0	M0	M0	M1	M1	M1	M3	M3	M3	M6	M6	M6

*Note:* Ca (mg/dL), PTH (pg/mL), and P (mg/dL).

The calcium and PTH dropped substantially for both PEA and PTX for one month and remained low for 6 months. There is no difference in values at 1, 3, and 6 months, suggesting that both PEA and PTX had similar effects on PTH and calcium after treatment. Therefore, both groups responded favorably. Notably, the PTH in the PEA group was higher at baseline, and although it decreased by about the same amount as the PTX group (80%), it remained higher. This was not statistically significant.

A summary of the number and percentage of patients with normal calcium, PTH, and phosphorus levels at each visit is provided in Table 3 for the ethanol ablation group and Table 4 for the PTX group. A chi‐square test was performed to compare these laboratory values before and after the procedure. Based on the *p* value < 0.01, both treatment methods have normalized the tests to a significant extent and have been effective except for the phosphorus levels.

**TABLE 3:** Lab values and percentages of calcium (Ca), parathormone (PTH), and phosphorus (P) in the percutaneous ethanol ablation (PEA) group at baseline and Months 1, 3, and 6.

PEA	M0	M1	M3	M6	Statistical analysis	*p* value
Normalized calcium number	0	9	8	8	Chi‐square test	< 0.001
Normalized calcium percent	0%	82%	73%	73%	Chi‐square test	< 0.001
Normalized PTH number	0	6	7	5	Chi‐square test	0.007
Normalized PTH percent	0%	55%	63%	45%	Chi‐square test	0.007
Normalized phosphorus number	10	8	8	9	Chi‐square test	0.845
Normalized phosphorus percent	9%	73%	73%	82%	Chi‐square test	0.845

**TABLE 4:** Lab values and percentages of calcium (Ca), parathormone (PTH), phosphorus (P) in the parathyroidectomy (PTX) group at baseline and Months 1, 3, and 6.

PTX	M0	M1	M3	M6	Statistical test	*p* value
Normalized calcium number	0	17	18	17	Chi‐square test	< 0.001
Normalized calcium percent	0%	85%	90%	85%	Chi‐square test	< 0.001
Normalized PTH number	0	14	15	17	Chi‐square test	< 0.001
Normalized PTH percent	0%	70%	75%	85%	Chi‐square test	< 0.001
Normalized phosphorus number	18	16	19	19	Chi‐square test	0.576
Normalized phosphorus percent	90%	84%	95%	95%	Chi‐square test	0.576

**FIGURE 3:**
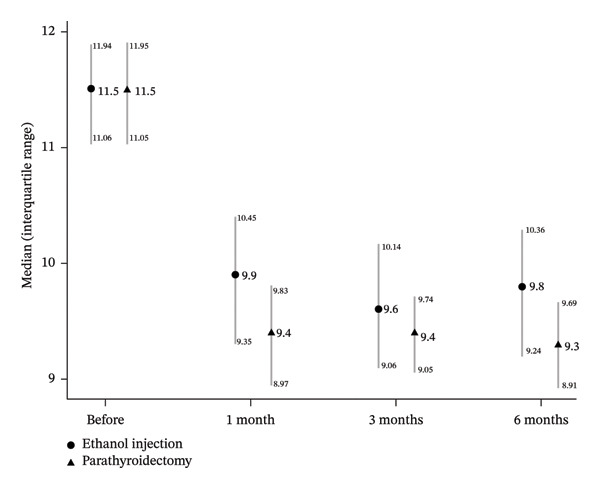
Calcium levels in different groups at different times.

**FIGURE 4:**
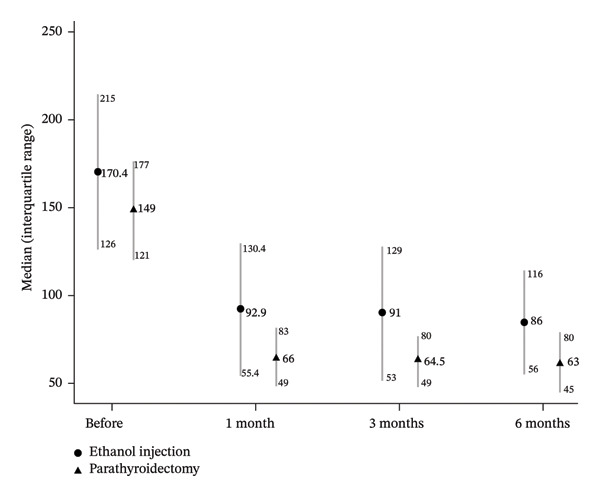
PTH levels in different groups at different times.

A longitudinal analysis was conducted to assess the impact of the two treatment methods on the reduction of calcium and PTH levels before and after the treatment procedure in each period. There was no significant difference between the laboratory values of the PTX and PEA after treatment, as indicated by a *p* value greater than 0.05. This suggests that ethanol injection and PTX were almost equally effective at reducing calcium and PTH levels (Figures 3 and 4). It should be noted that at baseline, the PTH values were 21 pg/mL higher in the PEA group. Although both groups experienced a drop in PTH of around 80 pg/mL, the PEA group remained higher throughout the study, albeit not statistically significant.

Six patients in the PEA group underwent a 12‐month follow‐up. All six had normal calcium levels in one year, and five (83.3%) had normal calcium and PTH levels. A 2‐year follow‐up was also possible in three patients with normal calcium and PTH levels. Patients with normalized hormone levels were considered cured.

Eight of the 31 patients studied experienced complications. We observed 2 cases of transient hoarseness in the PEA group. We also observed two cases of transient hoarseness, one case of permanent hoarseness, and three cases of symptomatic hypocalcemia in the PTX group. Laryngoscopy confirmed recurrent nerve injury causing hoarseness in both cases.

## 4. Discussion

Several minimally invasive treatment options have been available in recent years for hyperparathyroidism, including PEA. This study aimed to compare PEA with surgery, the current gold standard treatment method. Scientists attribute the therapeutic effect of ethanol to two mechanisms: (1) coagulative necrosis of the target tissue and (2) ischemic necrosis because of small blood vessel thrombosis.

A few studies have been conducted on the effectiveness of ethanol injections. Yankova et al. conducted a retrospective study on 35 patients undergoing PEA who had contraindications to surgery. In 3 months, 74.3% of patients had normal calcium levels, and 54.3% had normal PTH levels. Sixty‐two point eight percent had normal calcium levels within 6 months [20]. Several case reports have been written in which parathyroid adenoma was treated with ethanol, resulting in normal levels of calcium and PTH [21–24]. A retrospective study was conducted by Nakhle et al. on 37 patients. One to three months after injection, 63.5% of patients responded entirely, and 31.7% responded partially to treatment. Seventy‐two point two percent of patients remained eucalcemic 6 to 9 months after receiving the ethanol injection, and 62.2% remained eucalcemic 1 to 3 years later [25]. In a study by Yazdani et al., 39 patients with parathyroid adenoma and surgical contraindications were evaluated. As a result of ethanol injection, 46% of patients normalized within 1 month and 84.5% within 1 year [26]. Cercueil et al. observed normalization of calcium and PTH levels in 56% of patients [27].

Most studies do not include a control group, and only the normalization of the ethanol injection group was reported. Moreover, most patients who underwent this treatment method had contraindications for PTX due to comorbidities and were not randomly selected, which can negatively impact the study results. A more recent study has addressed these weaknesses. Eslamian et al. conducted a nonblind parallel clinical trial in patients with solitary parathyroid adenoma and high PTH. They assigned 68 patients to the PTX group and 68 to the PEA group. PTH, serum‐adjusted calcium, and adenoma size and volume were all significantly reduced by PTX and PEA, with no significant difference between them. They concluded that PEA is an effective alternative to PTX, particularly in adenomas with a volume of less than 425.5 mm^3^ and a maximum diameter of 13.5 mm [28].

A meta‐analysis by Bilgin et al. compared 800 cases of parathyroid adenomas treated with PEA, RF ablation, and MWA ablation but did not include the surgical option [29]. They concluded that PEA is safe and effective within the first year of the treatment and like RF and MWA [29].

We designed this study to eliminate the previously mentioned flaws and biases. Our patients were partially randomly assigned to two treatment groups based on patient preference, and the analysis showed no significant differences between the two groups in terms of age, gender, presence of underlying disease, and size of adenoma. Unfortunately, the 2020–22 pandemic significantly impacted our work, and we were unable to recruit as many candidates as we had hoped. This resulted in an underpower study, but we believe it still presents usable and practical information.

All cases in our study had a decrease in calcium and PTH levels following ethanol injection and surgery. After 6 months, 72.7% of patients had normal calcium levels, and 45.45% had normal PTH levels. There was no significant difference between the case and control groups (PEA vs. PTX) in calcium, phosphorus, and PTH levels. Ethanol ablation response was not related to the adenoma size or volume. Therefore, PEA was as effective as the gold standard for treating primary hyperparathyroidism. The advantages of PEA include its lower cost, milder side effects, shorter or no hospital stay, lack of anesthesia, the possibility of repeated treatment, and its suitability for individuals with underlying diseases and surgical contraindications.

According to other studies, this method can damage the recurrent laryngeal nerve and cause temporary or permanent hoarseness, hypocalcemia, and pain at the injection site. In this study, two patients experienced transient hoarseness after PEA. The PTX group also reported 2 cases of transient hoarseness, 1 case of permanent hoarseness, and 3 cases of hypocalcemia. Although statistically insignificant, these complications (especially the permanent voice change observed in PTX) play a role.

Due to the limited number of patients in our study, the power was insufficient. Power could have affected the outcomes. We also had other limitations, including the impact of the pandemic on patient selection. More patients opted for the surgical method, which might have skewed our results.

Although some nonstatistically significant differences were present, with PEA having less prominent effects but also fewer side effects; overall, the two groups were equal. However, our numbers were not as large as we would have hoped they would be.

A current challenge is the absence of clear guidelines to determine the appropriate interval between ethanol injections and between the PEA and laboratory tests. In addition, further studies with more participants (increasing the study’s power), better randomization, and longer follow‐ups are needed to shed more light on this subject. A comparison study among RFA, MWA, PEA, and PTX would be fascinating and might ultimately settle the debate.

## 5. Conclusion

In statistical terms, our team noted no difference between the PEA and PTX. Nonstatistically significant data suggest that PEA is less effective but safer than PTX.

## Funding

There was no funding or grants.

## Ethics Statement

We explained the ethical approval and consent in the Methods section.

## Conflicts of Interest

The authors declare no conflicts of interest.

## Data Availability

Data are available on request.

## References

[1] Lee K. H. and Jeong I. , Combination Therapy of Ethanol Ablation and Radiofrequency Ablation to Treat Parathyroid Adenoma in a Case With Primary Hyperparathyroidism, Iranian Journal of Radiology. (2022) 19, no. 4, 10.5812/iranjradiol-120869.

[2] Fraser W. D. , Hyperparathyroidism, Lancet.(2009) 374, no. 9684, 145–158, 10.1016/s0140-6736(09)60507-9. 19595349

[3] Grzela T. , Chudzinski W. , Lasiecka Z. et al., The Calcium-Sensing Receptor and Vitamin D Receptor Expression in Tertiary Hyperparathyroidism, International Journal of Molecular Medicine. (2006) 17, no. 5, 779–783, 10.3892/ijmm.17.5.779. 16596260

[4] Rasmuson T. and Tavelin B. , Risk of Parathyroid Adenomas in Patients With Thyrotoxicosis Exposed to Radioactive Iodine, Acta Oncologica. (2006) 45, no. 8, 1059–1061, 10.1080/02841860500516618. 17118839

[5] Kebebew E. , Duh Q.-Y. , and Clark O. H. , Tertiary Hyperparathyroidism: Histologic Patterns of Disease and Results of PTX, Archives of Surgery. (2004) 139, no. 9, 974–977, 10.1001/archsurg.139.9.974. 15381615

[6] Bilezikian J. P. , Bandeira L. , Khan A. , and Cusano N. E. , Hyperparathyroidism, Lancet. (2018) 391, no. 10116, 168–178, 10.1016/s0140-6736(17)31430-7. 28923463

[7] Bandeira F. , Griz L. , Chaves N. et al., Diagnosis and Management of Primary Hyperparathyroidism: A Scientific Statement From the Department of Bone Metabolism, the Brazilian Society for Endocrinology and Metabolism, Arquivos Brasileiros de Endocrinologia & Metabologia. (2013) 57, no. 6, 406–424, 10.1590/s0004-27302013000600002. 24030180

[8] Yu N. , Donnan P. T. , Murphy M. J. , and Leese G. P. , Epidemiology of Primary Hyperparathyroidism in Tayside, Scotland, UK, Clinical Endocrinology. (2009) 71, no. 4, 485–493, 10.1111/j.1365-2265.2008.03520.x. 19751296

[9] Gayed I. W. , Kim E. E. , Broussard W. F. et al., The Value of 99mTc-sestamibi SPECT/CT Over Conventional SPECT in the Evaluation of Parathyroid Adenomas or Hyperplasia, Journal of Nuclear Medicine. (2005) 46, no. 2, 248–252. 15695783

[10] Carty S. E. , Worsey M. J. , Virji M. A. , Brown M. L. , and Watson C. G. , Concise PTX: The Impact of Preoperative SPECT 99mTc Sestamibi Scanning and Intraoperative Quick Parathormone Assay, Surgery. (1997) 122, no. 6, 1107–1116, 10.1016/s0039-6060(97)90215-4. 9426426

[11] Udelsman R. , Lin Z. , and Donovan P. , The Superiority of Minimally Invasive PTX Based on 1650 Consecutive Patients With Primary Hyperparathyroidism, Annals of Surgery. (2011) 253, no. 3, 585–591, 10.1097/sla.0b013e318208fed9. 21183844

[12] Van Udelsman B. and Udelsman R. , PTXin Primary Hyperparathyroidism: Extensive Personal Experience, Journal of Clinical Densitometry. (2013) 16, no. 1, 54–59, 10.1016/j.jocd.2012.11.007. 23374742

[13] Marcus R. , Madvig P. , Crim M. , Pont A. , and Kosek J. , Conjugated Estrogens in the Treatment of Postmenopausal Women With Hyperparathyroidism, Annals of Internal Medicine. (1984) 100, no. 5, 633–640, 10.7326/0003-4819-100-5-633. 6324624

[14] Selby P. L. and Peacock M. , Ethinyl Estradiol and Norethindrone in the Treatment of Primary Hyperparathyroidism in Postmenopausal Women, New England Journal of Medicine. (1986) 314, no. 23, 1481–1485, 10.1056/nejm198606053142304. 3754618

[15] Yamashita T. , Mirande M. , Huang C. et al., Persistence and Recurrence of Hypercalcema After PTX over 5 Decades (1965–2010) in a Community-Based Cohort, Annals of Surgery. (August 1 2023) 278, no. 2, e309–e313. 36017920 10.1097/SLA.0000000000005688 PMC9968357

[16] Singh Ospina N. , Thompson G. B. , Lee R. A. , Reading C. C. , and Young W. F. , Safety and Efficacy of Percutaneous Parathyroid Ethanol Ablation in Patients With Recurrent Primary Hyperparathyroidism and Multiple Endocrine Neoplasia Type 1, Journal of Clinical Endocrinology & Metabolism. (2015) 100, no. 1, E87–E90, 10.1210/jc.2014-3255. 25337928

[17] Harman C. R. , Grant C. S. , Hay I. D. et al., Indications, Technique, and Efficacy of Alcohol Injection of Enlarged Parathyroid Glands in Patients With Primary Hyperparathyroidism, Surgery. (1998) 124, no. 6, 1011–1019, 10.1067/msy.1998.91826. 9854577

[18] Solbiati L. , Giangrande A. , De Pra L. , Bellotti E. , Cantù P. , and Ravetto C. , Percutaneous Ethanol Injection of Parathyroid Tumors Under US Guidance: Treatment for Secondary Hyperparathyroidism, Radiology. (1985) 155, no. 3, 607–610, 10.1148/radiology.155.3.3889999. 3889999

[19] Bennedbæk F. N. , Karstrup S. , and Hegedüs L. , Percutaneous Ethanol Injection Therapy in the Treatment of Thyroid and Parathyroid Diseases, European Journal of Endocrinology. (1997) 136, no. 3, 240–250, 10.1530/eje.0.1360240. 9100545

[20] Yankova I. , Shinkov A. , and Kovatcheva R. , Evaluation of the Early Results of Percutaneous Ethanol Ablation in Patients With Primary Hyperparathyroidism, Acta Medica Bulgarica. (2022) 49, no. 1, 5–11, 10.2478/amb-2022-0001.

[21] Soma R. , Takayama Y. , and Mimo T. , Changes in Serum Intact Parathyroid Hormone Levels and Ultrasonic Findings After Percutaneous Ethanol Injection Therapy in a Patient With Primary Hyperparathyroidism, Nihon Naibunpi Gakkai Zasshi. (1995) 71, no. 1, 53–64, 10.1507/endocrine1927.71.1_53. 7895865

[22] Akel M. , Salti I. , and Azar S. T. , Successful Treatment of Parathyroid Cyst Using Ethanol Sclerotherapy, American Journal of the Medical Sciences. (1999) 317, no. 1, 50–52, 10.1016/s0002-9629(15)40469-0. 9892271

[23] Halenka M. , Frysak Z. , Koranda P. et al., UltrasoUnd-gUided PercUtaneoUs Ethanol Injection Therapy in a 92 Year-Old Patient With Parathyroid Adenoma and With a History of Total Thyroidectomy for PaPillary Thyroid Carcinoma, Acta Endocrinologica. (2016) 12, no. 3, 349–354, 10.4183/aeb.2016.349. 31149113 PMC6535268

[24] Bansal S. , Kaushik R. , Modi S. , Raghuvanshi S. , and Kusum A. , Primary Hyperparathyroidism Presenting as Severe Hypercalcemia With Acute Pancreatitis in Pregnancy, Gynecological Endocrinology. (2020) 36, no. 5, 469–472, 10.1080/09513590.2019.1698028. 31793366

[25] Nakhle S. , Hoefler E. , Izkhkov N. , Ayutyanont N. , and Akhondi H. , OR30-2 Efficacy and Safety of Percutaneous Ethanol Ablation of Parathyroid Adenoma, Journal of the Endocrine Society. (2019) 3, no. Suppl 1, OR30–OR32, 10.1210/js.2019-or30-2.

[26] Yazdani A. A. , Khalili N. , Siavash M. et al., Ultrasound-Guided Ethanol Injection for the Treatment of Parathyroid Adenoma: A Prospective Self-Controlled Study, Journal of Research in Medical Sciences. (2020) 25, no. 1, 10.4103/jrms.jrms_553_19. PMC7698383 33273938

[27] Cercueil J. , Jacob D. , Verges B. , Holtzmann P. , Lerais J. M. , and Krause D. , Percutaneous Ethanol Injection Into Parathyroid Adenomas: Mid-and Long-Term Results, European Radiology. (1998) 8, no. 9, 1565–1569, 10.1007/s003300050587. 9866762

[28] Eslamian M. , Tavakoli B. , Firouzfar A. et al., Comparison of the Two Treatment Methods in Primary Hyperparathyroidism due to Solitary Parathyroid Adenoma, Ultrasound-Guided Percutaneous Alcohol Ablation vs. Parathyroidectomy: A Randomized Controlled Trial, BMC Endocrine Disorders. (2025) 25, no. 1, 10.1186/s12902-025-01836-0. PMC11761177 39849501

[29] Bilgin C. , Hibbert R. , Oztepe F. et al., Percutaneous Ablation of Parathyroid Adenomas: A Systematic Review and Meta-Analysis, Journal of Clinical Endocrinology & Metabolism. (May 2025) 6, no. 9, dgaf270–e3162, 10.1210/clinem/dgaf270. 40326768

